# Association of Multiple Hospital Affiliations With Clinician Service Use, Breadth of Procedures, and Costs

**DOI:** 10.1001/jamanetworkopen.2021.39169

**Published:** 2021-12-16

**Authors:** Sebastian Linde, Svetlana Beilfuss

**Affiliations:** 1 Division of General Internal Medicine, Department of Medicine, Medical College of Wisconsin, Milwaukee; 2 Center for Advancing Population Sciences, Medical College of Wisconsin, Milwaukee; 3 Department of Economics, Eastern Michigan University, Ypsilanti

## Abstract

**Importance:**

Little is known about whether a clinician having multiple hospital affiliations (ie, 1 clinician working across multiple teams and organizations) is associated with clinician practice style and cost. The measurement of this association requires adjusting for selection into multihospital affiliations based on both observable and unobservable clinician characteristics.

**Objective:**

To evaluate the association of multiple hospital affiliations with clinician service use, breadth of procedures used, and costs.

**Design, Setting, and Participants:**

This cohort study used Medicare Part B data from 2016 through 2017 in a fixed-effects panel data design to compare service use, procedure breadth, and costs between clinicians with multiple affiliations (treatment group) and clinicians with a single affiliation (control group), with adjustment for volume, patients, and clinician characteristics. The study also controlled for unobserved (time-invariant) clinician characteristics using individual clinician fixed effects. Clinicians with Medicare claims, a reported National Provider Identifier, and affiliation data within Medicare Physician Compare were included for a total sample of 1 073 252 observations (633 552 unique clinicians) for medical services and 358 669 observations (210 260 unique clinicians) for drug prescribing. Statistical analyses were performed from February 1 to October 15, 2021.

**Main Outcomes and Measures:**

Service use is the total number of medical (or drug) services that clinicians render to their Medicare beneficiaries within a given year, procedure breadth is the total number of unique Healthcare Common Procedure Coding System codes that are associated with clinicians’ medical (or drug) services within a given year, and costs represent the total standardized amount paid by Medicare for the medical (or drug) services. Additional measures were multiple-hospital affiliations, Accountable Care Organization affiliation, and controls across clinician and patient characteristics.

**Results:**

The medical service sample consisted of 633 552 clinicians (248 359 women [39.2%]; mean [SD] of 19.6 [12.5] years of experience), and the drug service sample consisted of 210 260 clinicians (74 875 women [35.6%]; mean [SD] of 21.6 [12.3] years of experience). For medical services, clinicians with multiple practice affiliations used a mean 8.2% (95% CI, 7.5%-8.9%; *P* < .001) more medical services per patient, drew on a mean 5.4% (95% CI, 5.1%-5.7%; *P* < .001) wider set of procedures within their medical care, and incurred a mean 8.6% (95% CI, 7.9%-9.2%; *P* < .001) more in medical costs. Pertaining to drug services, clinicians with multiple practice affiliations used a mean 2.9% (95% CI, 1.9%-3.9%; *P* < .001) more drug services per patient, drew on a mean 1.0% (95% CI, 0.5%-1.4%; *P* < .001) wider set of procedures within their medical care, and incurred a mean 2.7% (95% CI, 1.6%-3.7%; *P* < .001) more in drug costs. Significant results were also found across extensive and intensive margins of hospital affiliation, and supplemental analysis further indicated heterogenous treatment associations across clinician specialties.

**Conclusions and Relevance:**

This cohort study found that a clinician having multihospital affiliations was associated with greater service use, procedure breadth, and costs across both medical and drug services. These findings suggest that clinician affiliations ought to be considered as part of health care delivery design and potential cost-containment strategies.

## Key Points

**Question:**

Is a clinician having multiple hospital affiliations (ie, 1 clinician working across multiple teams and organizations) associated with clinician practice style and cost?

**Findings:**

This cohort study found that, with adjustment for both observable and unobservable (time-invariant) clinician characteristics, clinicians with multiple hospital affiliations used a mean 8.2% more medical services per patient, drew on a mean 5.4% wider set of procedures within their medical care, and incurred a mean 8.6% more in medical costs. These findings are statistically significant, as are both extensive and intensive margin associations; the study further found evidence of heterogenous treatment responses across clinician specialties.

**Meaning:**

These findings suggest that clinician affiliations ought to be considered as part of health care delivery design and cost-containment strategies.

## Teaser

This cohort study uses Medicare Part B data to evaluate the association of multiple hospital affiliations with clinician service use, breadth of procedures used, and costs.

## Introduction

In recent years, several trends have been associated with a shift in health care clinicians increasing their institutional affiliations. Both an increase in physician-hospital integration and the rollout of new innovative payment models, such as Accountable Care Organizations (ACOs), have been shown to alter affiliated clinicians’ practice styles.^1,2,3,4^ The notion that clinicians’ practice patterns vary across hospital and practice settings, even within smaller geographical areas^5,6,7,8,9^ and with the resource accessibility of clinicians,^10,11^ has had longstanding support within the literature. Prior work has further noted important associations between a clinician having multiple hospital affiliations (ie, 1 clinician seeing patients across multiple hospitals and teams) and medical costs.^12,13,14^ However, these previously estimated associations may be susceptible to bias if health care clinicians self-select into being affiliated with multiple hospitals based on both observable and unobservable clinician characteristics that also may influence their practice styles. As such, accounting for unobserved clinician confounders presents an important obstacle for obtaining appropriate estimates of the association of a clinician having multiple hospital affiliations with clinician practice style, use of services and procedures, and costs.

In theory, the direction of the association of multihospital affiliations is not clear. On one hand, more clinician affiliations across hospitals may increase coordination problems and limit clinician availability to patients, inducing multihospital clinicians to compensate for the lack of continuity in care by engaging in defensive medicine and by increasing their reliance on diagnostic tests and support staff rather than thorough patient examination.^12,13,15^ Greater use of hospital resources may then lead to increased costs.^1^ On the other hand, more hospital affiliations may improve care and lower costs by facilitating the transmission of new medical knowledge, best-care practices, and greater access to electronic health records.^16,17,18,19^

In this study, we examine how multiple hospital affiliations are associated with clinicians’ use of medical services, the variety of procedures undertaken, and clinician-generated costs using recent Medicare data from 2016 and 2017. Our use of a panel data set allows us to estimate these associations with adjustment for selection into multihospital affiliations based on both observable and unobservable (time-invariant) clinician characteristics.

## Methods

### Study Sample

In this cross-sectional study, we analyzed a panel of clinicians (primary care clinicians, medical specialists, surgical specialists, and midlevel practitioners) from the 2016-2017 Medicare Fee-for-Service Provider Utilization and Payment Data (the Physician and Other Supplier Public Use File). This database contains 100% of all final-action claims information on Medicare Part B services and procedures provided by health care clinicians to Medicare beneficiaries enrolled in the fee-for-service program.^20^ Using National Provider Identifiers and year, we further linked these data with the Medicare Physician Compare data set that contains up-to-date information on clinicians and groups enrolled in Medicare.^21^ From the Medicare Physician Compare data set, we obtained the demographic characteristics of the clinicians, as well as their hospital and Medicare ACO affiliations. This study is based on publicly available deidentified data and it did not constitute human subjects research as defined by 45 CFR §46.102. This study follows the Strengthening the Reporting of Observational Studies in Epidemiology (STROBE) reporting guidelines.

Drawing on these sources, we constructed 2 data samples—one comprising medical service clinicians and the other including drug service clinicians (ie, clinicians who write prescriptions). The medical service clinician sample for 2016-2017 consisted of 1 073 252 observations (633 552 clinicians, most of whom are in both years of the data), while the drug service sample had 358 669 observations (210 260 unique clinicians). Detailed primary specialty breakdowns can be seen in eTables 1 and 2 in the Supplement.

### Study Variables

#### Outcome Measures: Services, Procedure Breadth, and Cost

Our outcome measures span medical and drug services, procedure breadth, and costs. These are defined as follows. First, the medical service measure is the total number of medical services that a clinician renders to their Medicare beneficiaries within a given year. Similarly, the drug service measure captures the total number of drug services rendered per the definition of the Medicare Part B Drug Average Sale Price File.^22^

Second, the breadth of medical procedures is given by the total number of unique Healthcare Common Procedure Coding System codes that are associated with a clinician’s medical services within a given year. The breadth of drug procedures is similarly given by the total number of unique drug service Healthcare Common Procedure Coding System codes.

Last, medical costs are defined by the total standardized amount paid by Medicare (after the deductible and coinsurance amounts have been deducted) for the medical service. Standardized payments refer to payments that account for geographical differences in payment rates for individual services, and as such, variation within these payments are said to “reflect variation in factors such as physicians’ practice patterns and beneficiaries’ ability and willingness to obtain care.”^20^^(p10)^ The standardized amount paid by Medicare for drug services, which we refer to as our *drug costs outcome*, is equivalently defined.

#### Exposure Measure: Clinician Affiliation

Our main exposure measure was a multihospital affiliation indicator variable that takes the value of 1 for multihospital-affiliated (treated) clinicians and 0 for single-hospital–affiliated (control) clinicians. To explore extensive- and intensive-margin associations, we also used an additional main exposure measure—the total number of hospital affiliations that a given Medicare clinician has. Extensive-margin results capture the association of a clinician changing affiliation status from no hospital affiliation to a hospital affiliation. Intensive-margin results capture the incremental association of a clinician moving from having a positive number of hospital affiliations to having 1 more affiliation. These variables are constructed using the Medicare Physician Compare database, within which a clinician is coded as being affiliated with a hospital if they have (within a given year) billed Medicare for at least 3 different patients, on 3 different occasions (dates), from the hospital in question. In addition to our main hospital affiliation measures, we also controlled for whether a clinician was affiliated with at least 1 Medicare ACO (ie, across the Medicare Shared Savings Program, ACO Investment Model, Advance Payment Model, Next Generation ACO Model, Pioneer ACO Model, Vermont All-Payer ACO Model, and Comprehensive ESRD Care Initiative).

#### Other Covariates

Additional controls were included across clinician and patient pool characteristics. On the clinician side, we controlled for years of experience that the clinician has (measured as a count of the years since they graduated from medical school) and whether or not they used electronic health records. Regarding the clinicians’ patient pool, we controlled for (1) the beneficiaries’ mean Hierarchical Condition Category risk score, (2) the mean age of the Medicare beneficiaries seen by the clinician (patient age is calculated at the end of the calendar year or at the time of death), (3) the number of female beneficiaries, and (4) the number of unique beneficiaries that a clinician has provided medical (or drug) services to. These variables account for any differences in clinicians’ patient populations that are associated with the cost and the types of procedure performed by the clinician, and that may also be associated with affiliation choices.

### Statistical Analysis

Statistical analyses were performed from February 1 to October 15, 2021. We used panel data regression methods to estimate the association of clinician hospital affiliations with service breadth, procedure breadth, and costs. On account of the inherent limitations of observational study designs, we took the following precautions to decrease concerns of potential confounding due to unobserved variables. First, we controlled for a rich set of clinician, patient pool, and volume features. Second, by using a fixed-effects specification, we controlled for clinician and year fixed effects—these allowed us to control for any unobserved (time-invariant) clinician (or year) differences that might be associated with our hospital affiliation measure as well as our outcome measures. As such, our specification ameliorated concerns pertaining to clinician selection into multihospital affiliations based on either observable or unobserved clinician characteristics that are time-invariant. The details of our regression model specification can be found in eAppendix 1 in the Supplement. Last, we used the multihospital affiliation indicator as our central exposure measure within our main analyses. We evaluated statistical significance at 3 levels of significance within the study: *P* < .01, *P* < .05, and *P* < .10; hypotheses tests were 2-sided. All analyses were performed using Stata MP, version 17 (StataCorp LLC).

### Supplemental Analyses and Sensitivity Checks

First, as a sensitivity check, we examined whether the results were qualitatively similar when the clinician’s number of hospital affiliations was used as our main exposure variable instead. Second, we examined whether the number of measured associations for this measure (ie, the number of hospital affiliations) was monotonically increasing. This was accomplished by estimating a model in which the number of hospital affiliations was also entered as a quadratic term. Third, we also examined whether the factors associated were multihospital affiliations were heterogenous across different medical specialties. This was done by including specialty level and interaction terms for clinicians with a primary specialty of (1) internal medicine, (2) family or general practice, or (3) being a nurse practitioner.

## Results

### Medical Service and Drug Service Samples

The medical service sample consisted of 633 552 clinicians (248 359 women [39.2%]; mean [SD] of 19.6 [12.5] years of experience) and the drug service sample consisted of 210 260 clinicians (74 875 women [35.6%]; mean [SD] of 21.6 [12.3] years of experience). Table 1 shows summary statistics for our outcome, affiliation, and control measures across both the medical service and the drug service samples. The mean (SD) number of affiliations within our sample (which is based on the condition that the clinicians are affiliated with at least 1 hospital) is 2.2 (1.4) for the medical service sample and 2.6 (1.4) for the drug service sample. Of all the clinicians, a mean (SD) 58% (49%) were affiliated with multiple hospitals within the medical sample and 70% (46%) were affiliated with multiple hospitals within the drug sample, while 28% (45%) were affiliated with ACOs within the medical sample and 29% (46%) were affiliated with ACOs within the drug sample.

**Table 1.** Summary Statistics (2016-2017) for Outcome Measures, Affiliation Measures, and Control Variables, When the Clinician Has at Least 1 Hospital Affiliation

Variable	Mean (SD) value	
	Medical services sample	Drug services sample
Medical outcome measures		
Medical services, No.	1513.62 (3021.30)	NA
Medical procedures breadth, No.	40.59 (35.88)	NA
Total medical costs, $	90 041.09 (135 497.11)	NA
Drug outcome measures		
Drug services, No.	NA	5479.23 (28 134.64)
Drug procedures breadth, No.	NA	7.97 (9.85)
Total drug costs, $	NA	62 264.06 (310 844.96)
Affiliation measures		
Hospital affiliations, No.	2.2 (1.4)	2.6 (1.4)
Multihospital affiliations, %	58 (49)	70 (46)
ACO affiliated, %	28 (45)	29 (46)
Control variables		
Experience, y	20.5 (12.3)	22.6 (12.1)
EHR user, %	30 (46)	49 (50)
Mean risk score	1.78 (0.80)	1.42 (0.57)
Mean patient age, y	71.1 (5.1)	72.2 (3.7)
No. of female patients	250.39 (393.07)	307.69 (387.02)
No. of medical patients	439.43 (661.58)	NA
No. of drug patients	NA	98.44 (103.84)
No. of observations	1 073 252	358 669

Abbreviations: ACO, Accountable Care Organization; EHR, electronic health record; NA, not applicable.

Table 2 shows the same descriptive statistics by clinician affiliation status contrasted with single-hospital and multihospital affiliations. In the medical service sample, multihospital-affiliated clinicians provided 1983.41 services and single-hospital–affiliated clinicians provided 872.15 services, for a difference of 1111.26 more services (127.4%) provided by multihospital-affiliated clinicians. Multihospital-affiliated clinicians used 47.42 procedures and single-hospital–affiliated clinicians used 31.25 procedures, for a difference of 16.17 additional types of procedures (51.7%) provided by multihospital-affiliated clinicians. Multihospital-affiliated clinicians incurred $115 165.28 in medical costs and single-hospital–affiliated clinicians incurred $55 734.60 in medical costs, for a difference of $59 430.68 more in medical costs (106.6%) incurred by multihospital-affiliated clinicians. The drug service sample shows similar trends, with multihospital-affiliated clinicians providing 6792.2 drug services and single-hospital–affiliated clinicians providing 2382.0 drug services, for a difference of 4410.2 more drug services (185.1%) provided by multihospital-affiliated clinicians. Multihospital-affiliated clinicians used 8.38 procedures and single-hospital–affiliated clinicians used 7.00 procedures, for a difference of 1.38 more procedures (19.7%) provided by multihospital-affiliated clinicians. Multihospital-affiliated clinicians incurred $69 668.76 in drug costs and single-hospital–affiliated clinicians incurred $44 802.32 in drug costs, for a difference of $24 866.44 more in drug costs (55.5%) incurred by multihospital-affiliated clinicians.

**Table 2.** Summary Statistics (2016-2017) for Outcome Measures, Affiliation Measures, and Control Variables

Variable	Medical services sample		Drug services sample	
	Single hospital affiliation	Multihospital affiliations	Single hospital affiliation	Multihospital affiliations
Medical outcome measures				
Medical services, No.	872.15	1983.41	NA	NA
Medical procedures breadth, No.	31.25	47.42	NA	NA
Total medical costs, $	55 734.60	115 165.28	NA	NA
Drug outcome measures				
Drug services, No.	NA	NA	2383.00	6792.20
Drug procedures breadth, No.	NA	NA	7.00	8.38
Total drug costs, $	NA	NA	44 802.32	69 668.76
Affiliation measures				
Hospital affiliations, No.	1	3.11	1	3.21
Multihospital affiliations, %	0	1	0	1
ACO affiliated, %	26	29	28	30
Control variables				
Experience, y	19.1	21.6	20.6	23.4
EHR user, %	21	36	40	54
Mean risk score	1.78	1.78	1.32	1.46
Mean patient age, y	70.2	71.7	71.8	72.4
No. of female patients	148.54	324.98	223.99	343.18
No. of medical patients	259.86	570.94	NA	NA
No. of drug patients	NA	NA	76.17	107.88
No. of observations	453 715	619 537	106 804	251 865

Abbreviations: ACO, Accountable Care Organization; EHR, electronic health records; NA, not applicable.

### Multiple Hospital Affiliations and Medical and Drug Services, Procedure Breadth, and Cost

Table 3 presents the fixed-effects regression results across our medical and drug outcome measures when we used the multihospital affiliation indicator as the main exposure variable. Among the medical service sample, even after controlling for clinician and patient pool characteristics, as well as risk scores and clinician fixed effects, the services provided per patient among multihospital-affiliated clinicians were 8.2% (95% CI, 7.5%-8.9%; *P* < .001) higher than single-hospital–affiliated clinicians. In addition, multihospital-affiliated clinicians had a medical procedure breadth that is 5.4% (95% CI, 5.1%-5.7%; *P* < .001) greater than single-hospital clinicians; multihospital-affiliated clinicians also incurred 8.6% (95% CI, 7.9%-9.2%; *P* < .001) higher costs per patient than single-hospital clinicians. Qualitatively, the results from the drug service sample are similar. Multihospital-affiliated clinicians provided a mean of 2.9% (95% CI, 1.9%-3.9%; *P* < .001) more drug services, used a mean 1.0% (95% CI, 0.5%-1.4%; *P* < .001) broader set of drug procedures, and incurred a mean of 2.7% (95% CI, 1.6%-3.7%; *P* < .001) higher costs per patient than single-hospital–affiliated clinicians. eAppendix 1 and eTable 3 in the Supplement further show that using an alternative Poisson fixed-effects model was associated with qualitatively similar results to those reported here. To explore the association of hospital affiliation more broadly, we next examined the extensive and intensive margins of the measured hospital affiliation associations.

**Table 3.** Regression Estimates Across Medical Outcome Measures

Affiliation measure	Log-transformed value (95% CI)					
	Medical services sample			Drug services sample		
	Service use	Procedure breadth	Total cost	Service use	Procedure breadth	Total cost
Multihospital affiliations	0.082 (0.075 to 0.089)^a^	0.054 (0.051 to 0.057)^a^	0.086 (0.079 to 0.092)^a^	0.029 (0.019 to 0.039)^a^	0.010 (0.005 to 0.014)^a^	0.027 (0.016 to 0.037)^a^
ACO affiliation	0.002 (−0.001 to 0.005)	0.011 (0.009 to 0.013)^a^	0.001 (−0.003 to 0.004)	0.004 (−0.005 to 0.012)	0.008 (0.004 to 0.012)^a^	0.012 (0.002 to 0.021)^b^
Controls	Yes	Yes	Yes	Yes	Yes	Yes
Clinician FEs	Yes	Yes	Yes	Yes	Yes	Yes
Year FEs	Yes	Yes	Yes	Yes	Yes	Yes
No. of observations	1 073 252	1 073 252	1 073 252	358 669	358 669	358 669
*R* ^2^	0.191	0.052	0.163	0.202	0.041	0.221
No. of clinicians	633 552	633 552	633 552	210 260	210 260	210 260

Abbreviations: ACO, Accountable Care Organization; FE, fixed effects.

^a^ *P* < .001.

^b^ *P* < .05.

### Multiple Hospital Affiliations and Medical Services, Procedure Breadth, and Cost (Extensive and Intensive Margins)

Table 4 shows 2 sets of results. The first set shows the extensive margin of comparing clinicians with no hospital affiliation with those with a single hospital affiliation on the service, procedure breadth, and cost measures. The second set of results shows the intensive-margin estimates resulting from an incremental increase in hospital affiliations, based on the condition of at least 1 hospital affiliation (this differs from our previous results in that this association is measured using a hospital count measure rather than the multihospital indicator previously used in Table 3). The extensive-margin results show that increasing from no hospital affiliation to 1 hospital affiliation yields an increase in per-patient services of 8.7% (95% CI, 7.6%-9.7%; *P* < .001), an increased procedure breadth of 7.8% (95% CI, 7.2%-8.4%; *P* < .001), and an increase in per-patient cost of 9.2% (95% CI, 8.1%-10.2%; *P* < .001) (Table 4).

**Table 4.** Regression Estimates Across Medical Outcome Measures

Affiliation measure	Log-transformed value (95% CI)					
	0 or 1 Hospital affiliation			≥1 Hospital affiliations		
	Service use	Procedure breadth	Total cost	Service use	Procedure breadth	Total cost
Hospital affiliations	0.087 (0.076 to 0.097)^a^	0.078 (0.072 to 0.084)^a^	0.092 (0.081 to 0.102)^a^	0.049 (0.042 to 0.056)^a^	0.028 (0.026 to 0.030)^a^	0.052 (0.045 to 0.058)^a^
ACO affiliation	0.006 (0.001 to 0.012)^b^	0.010 (0.006 to 0.014)^a^	0.004 (−0.001 to 0.010)	0.001 (−0.002 to 0.004)	0.011 (0.008 to 0.013)^a^	−0.000 (−0.003 to 0.003)
Controls	Yes	Yes	Yes	Yes	Yes	Yes
Clinician FEs	Yes	Yes	Yes	Yes	Yes	Yes
Year FEs	Yes	Yes	Yes	Yes	Yes	Yes
No. of observations	785 437	785 437	785 437	1 073 252	1 073 252	1 073 252
*R* ^2^	0.140	0.029	0.131	0.195	0.054	0.167
No. of clinicians	509 564	509 564	509 564	633 552	633 552	633 552

Abbreviations: ACO, Accountable Care Organization; FE, fixed effects.

^a^ *P* < .001.

^b^ *P* < .05.

The intensive-margin results show that incremental increases in hospital affiliations yielded further increases across all 3 outcome measures (Table 4). Medical services increased by 4.9% (95% CI, 4.2-5.6%; *P* < .001) per additional affiliation, procedure breadth increased by 2.8% (95% CI, 2.6%-3.0%; *P* < .001) per additional affiliation, and medical cost increased by 5.2% (95% CI, 4.5%-5.8%; *P* < .001) per patient with each additional hospital affiliation.

### Multiple Hospital Affiliations and Drug Services, Procedure Breadth, and Cost (Extensive and Intensive Margins)

Table 5 presents extensive- and intensive-margin results for the drug service sample. The estimated extensive-margin coefficients show that increasing from no hospital to 1 hospital affiliation is associated with an increase in drug services of 5.4% (95% CI, 3.4%-7.5%; *P* < .001), an increase in drug procedure breadth of 2.2% (95% CI, 1.3%-3.1%; *P* < .001), and a mean increase in total drug costs of 5.3% (95% CI, 2.9%-7.7%; *P* < .001) per patient.

**Table 5.** Regression Estimates Across Drug Outcome Measures

Affiliation measure	Log-transformed value (95% CI)					
	0 or 1 Hospital affiliation			≥1 Hospital affiliations		
	Service use	Procedure breadth	Total cost	Service use	Procedure breadth	Total cost
Hospital affiliations	0.054 (0.034 to 0.075)^a^	0.022 (0.013 to 0.031)^a^	0.053 (0.029 to 0.077)^a^	0.017 (0.013 to 0.022)^a^	0.004 (0.002 to 0.007)^a^	0.015 (0.010 to 0.019)^a^
ACO affiliation	0.019 (0.001 to 0.037)^b^	0.010 (0.002 to 0.018)^b^	0.024 (0.005 to 0.043)^b^	0.003 (−0.005 to 0.012)	0.008 (0.004 to 0.012)^a^	0.011 (0.002 to 0.020)^b^
Controls	Yes	Yes	Yes	Yes	Yes	Yes
Clinician FEs	Yes	Yes	Yes	Yes	Yes	Yes
Year FEs	Yes	Yes	Yes	Yes	Yes	Yes
No. of observations	160 783	160 783	160 783	358 669	358 669	358 669
*R* ^2^	0.183	0.040	0.162	0.202	0.041	0.221
No. of clinicians	110 529	110 529	110 529	210 260	210 260	210 260

Abbreviations: ACO, Accountable Care Organization; FE, fixed effects.

^a^ *P* < .001.

^b^ *P* < .05.

The intensive-margin results are again qualitatively similar (Table 5). For a 1-unit increase in hospital affiliations, drug services increased by 1.7% (95% CI, 1.3%-2.2%; *P* < .001) per patient, drug procedure breadth increased by 0.4% (95% CI, 0.2%-0.7%; *P* < .001), and per-patient drug costs increased by 1.5% (95% CI, 1.0%-1.9%; *P* < .001).

### Supplemental Analyses and Sensitivity Checks

In supplementary analyses, we further found that the estimated multihospital affiliation associations are (1) robust to the use of an alternative hospital affiliation count measure (eTables 4-7 in the Supplement); (2) mostly nonmonotonic, exhibiting associations that are increasing (but at a decreasing rate) in the number of hospital affiliations (eTable 8 in the Supplement); and (3) heterogenous across different medical specialties (eAppendix 2 and eTables 9-11 in the Supplement). Last, using an alternative pooled regression analysis identification strategy resulted in qualitatively similar results (eTable 12 in the Supplement).

## Discussion

This cross-sectional study found that a clinician having multiple hospital affiliations was associated with increased clinician service use, greater procedure breadth, and higher costs across both medical and drug services. These associations are based on the adjustment for both observable clinician (and patient pool) characteristics and unobserved (time-invariant) clinician characteristics. As such, our findings suggest that clinician affiliations might be important to consider as part of health care delivery design and potential cost-containment strategies.

We found that both medical and drug (per-patient) services, as well as the variety of services used, were positively associated with multihospital affiliations. These findings suggest that clinicians may shift their treatment patterns—both treatment mix and quantity—when they start working across more hospitals. Because we controlled for the number and the health of Medicare beneficiaries, the estimated increase in services is unlikely to stem from patient volume or morbidity increases that may occur as clinicians become affiliated with more hospitals. Our specification also included variables for clinician ACO participation and electronic health record use, allowing us to control for possible confounding that may result from clinician differences in coordination levels and access to patient information.

Because hospitals generally offer a wider variety of services than clinician practices, it is possible that clinicians responded to this greater hospital resource accessibility by shifting patient procedures and diagnostic tests to the hospital outpatient departments after becoming affiliated. Associated with this increase in the breadth of procedures, the use of drugs is also likely to become more diverse as a result. Furthermore, in addition to wider resource accessibility, hospital settings enable affiliated clinicians to have greater access to other hospital-affiliated peers, thus promoting transmission of medical knowledge and best-care practices. Such peer relationships may also be associated with some of the increase in the breadth of services and drugs offered by affiliated clinicians. Thus, on the one hand, an increase in affiliations increases accessibility to a variety of treatment options and may be associated with more individualized and, potentially, better treatment strategies. On the other hand, some services may prove to be more cost-effective than others. Therefore, greater use of costlier hospital resources, whether as a result of defensive medicine or other hospital incentives, is potentially responsible for an increase in per-patient care use and costs of affiliated clinicians.

Furthermore, the finding that per-patient spending increased after controlling for the number and the variety of clinician-offered services suggests that clinicians may be using costlier hospital services as their hospital affiliations increase. It is also possible that clinicians start to shift their billing to hospital outpatient departments when they become affiliated with more hospitals because Medicare’s clinician-based billing policy creates an incentive for physicians to move services from office to outpatient settings where they are reimbursed at a higher rate. Further work investigating how much of the change in practice patterns is due to greater hospital resource availability and how much of it is due to clinicians being increasingly stretched for time, as they provide care across multiple hospitals, is warranted. In addition, the examination of potential heterogeneities across different types of contractual affiliations between clinicians and hospitals may also appear to present an important avenue for future research, as does a closer study of how policies may incorporate insights from this line of work.

### Limitations

There are 3 main limitations to our observational cross-sectional study. First, we note that our study outcomes may not be generalizable beyond the Medicare fee-for-service clinician population. Second, while our identification strategy is able to adjust for unobserved time-invariant features, our estimates are still sensitive to confounding from unobserved time-varian clinician features not controlled for within our study. Third, our results are also sensitive to confounding from unobserved patient and hospital heterogeneities.

## Conclusions

In the context of the present-day health care industry, increasing physician-hospital vertical integration trends are likely to increase clinicians’ affiliations with hospitals. However, most studies analyzing the increasing vertical integration between clinicians have been unable to control for potentially confounding factors and have shown little consensus in findings.^3^ Moreover, few studies use a more granular, clinician-level analysis, and, to our knowledge, none have examined the factors associated with extensive and intensive margins while accounting for potential selection into multihospital affiliations based on both observable and unobservable (time-invariant) clinician characteristics. Our findings indicate that, on average, being affiliated with a hospital is associated with increased service use, procedure breadth, and spending (ie, a significant extensive-margin association); also, we found that each additional hospital affiliation is continually associated with increased service use, procedure breadth, and costs (ie, a significant intensive-margin association). These results are not associated with unobserved (time-invariant) differences between clinicians with low vs high levels of hospital affiliations because our fixed-effects design allowed us to account for these differences and to identify our associations using only within-clinician variation within our data.

## Notes

Supplement. **eAppendix 1.** Statistical Analysis Additional Details **eTable 1.** Clinicians Primary Specialty Tabulations for the Medical Service Sample **eTable 2.** Clinicians Primary Specialty Tabulations for the Drug Service Sample **eTable 3.** Poisson Model Estimates Across Count Outcome Measure **eTable 4.** Regression Estimates Across Count Outcome Measure, With Quadratic Term Included to Evaluate Monotonicity of Association **eTable 5.** Regression Estimates Across Medical Outcome Measure **eTable 6.** Regression Estimates Across Drug Outcome Measure **eTable 7.** Summary Statistics for Outcome Measures, Affiliation Measures, and Control Variables Across Medical Services Sample, and Drug Services Sample **eTable 8.** Summary Statistics for Outcome Measures, Across Medical and Drug Service Samples, by Number of Clinician Hospital Affiliations **eAppendix 2.** Heterogenous Treatment Effects **eTable 9.** Heterogenous Treatment Association Estimates Across Internal Medicine (IM), Family/General Practice (FP) and Nurse Practitioners (NP) When Using the Dichotomous Multihospital Affiliation Measure **eTable 10.** Heterogenous Treatment Association Estimates Across Internal Medicine (IM), Family/General Practice (FP) and Nurse Practitioners (NP) for the Extensive and Intensive Margin Results for Medical Services **eTable 11.** Heterogenous Treatment Association Estimates Across Internal Medicine (IM), Family/General Practice (FP) and Nurse Practitioners (NP) for the Extensive and Intensive Margin Results for Drug Services **eTable 12.** Pooled Regression Estimates Across Medical Outcome Measure

## References

[1] Jung J, Feldman R, Kalidindi Y. The impact of integration on outpatient chemotherapy use and spending in Medicare. Health Econ. 2019;28(4):517-528. doi:10.1002/hec.3860 30695812 PMC6405302

[2] Koch TG, Wendling BW, Wilson NE. How vertical integration affects the quantity and cost of care for Medicare beneficiaries. J Health Econ. 2017;52:19-32. doi:10.1016/j.jhealeco.2016.12.007 28182998

[3] Machta RM, Maurer KA, Jones DJ, Furukawa MF, Rich EC. A systematic review of vertical integration and quality of care, efficiency, and patient-centered outcomes. Health Care Manage Rev. 2019;44(2):159-173. doi:10.1097/HMR.0000000000000197 29613860

[4] McWilliams JM, Chernew ME, Landon BE, Schwartz AL. Performance differences in year 1 of pioneer accountable care organizations. N Engl J Med. 2015;372(20):1927-1936. doi:10.1056/NEJMsa1414929 25875195 PMC4475634

[5] Egede LE, Gebregziabher M, Hunt KJ, . Regional, geographic, and ethnic differences in medication adherence among adults with type 2 diabetes. Ann Pharmacother. 2011;45(2):169-178. doi:10.1345/aph.1P442 21304026

[6] Fisher ES, Wennberg DE, Stukel TA, Gottlieb DJ, Lucas FL, Pinder ÉL. The implications of regional variations in Medicare spending, part 1: the content, quality, and accessibility of care. Ann Intern Med. 2003;138(4):273-287. doi:10.7326/0003-4819-138-4-200302180-00006 12585825

[7] Fisher ES, Wennberg DE, Stukel TA, Gottlieb DJ, Lucas FL, Pinder ÉL. The implications of regional variations in Medicare spending, part 2: health outcomes and satisfaction with care. Ann Intern Med. 2003;138(4):288-298. doi:10.7326/0003-4819-138-4-200302180-00007 12585826

[8] Walker RJ, Neelon B, Davis M, Egede LE. Racial differences in spatial patterns for poor glycemic control in the southeastern United States. Ann Epidemiol. 2018;28(3):153-159. doi:10.1016/j.annepidem.2018.01.008 29398299 PMC5828989

[9] Wennberg J, Gittelsohn A. Small area variations in health care delivery. Science. 1973;182(4117):1102-1108. doi:10.1126/science.182.4117.1102 4750608

[10] Baker LC. Acquisition of MRI equipment by doctors drives up imaging use and spending. Health Aff (Millwood). 2010;29(12):2252-2259. doi:10.1377/hlthaff.2009.1099 21134927

[11] Wennberg JE. Dealing with medical practice variations: a proposal for action. Health Aff (Millwood). 1984;3(2):6-32. doi:10.1377/hlthaff.3.2.6 6432667

[12] Miller ME, Welch WP, Welch HG. The impact of practicing in multiple hospitals on physician profiles. Med Care. 1996;34(5):455-462. doi:10.1097/00005650-199605000-00007 8614167

[13] Pauly MV. Medical staff characteristics and hospital costs. J Hum Resour. 1978;13(suppl):77-111. doi:10.2307/145249 722072

[14] Fisher ES, Staiger DO, Bynum JPW, Gottlieb DJ. Creating accountable care organizations: the extended hospital medical staff. Health Aff (Millwood). 2007;26(1):w44-w57. 17148490 10.1377/hlthaff.26.1.w44 PMC2131738

[15] Sicotte C, Pineault R, Lambert J. Medical team interdependence as a determinant of use of clinical resources. Health Serv Res. 1993;28(5):599-621. 8270423 PMC1069966

[16] Atasoy H, Greenwood BN, McCullough JS. The digitization of patient care: a review of the effects of electronic health records on health care quality and utilization. Annu Rev Public Health. 2019;40(1):487-500. doi:10.1146/annurev-publhealth-040218-044206 30566385

[17] Atasoy H, Chen P, Ganju K. The spillover effects of health IT investments on regional healthcare costs. Manage Sci. 2017;64(6):2515-2534. doi:10.1287/mnsc.2017.2750

[18] Kruse CS, Stein A, Thomas H, Kaur H. The use of electronic health records to support population health: a systematic review of the literature. J Med Syst. 2018;42(11):214. doi:10.1007/s10916-018-1075-6 30269237 PMC6182727

[19] Linde S. The formation of physician patient sharing networks in Medicare: exploring the effect of hospital affiliation. Health Econ. 2019;28(12):1435-1448. doi:10.1002/hec.3936 31657506 PMC6899902

[20] Centers for Medicare & Medicaid Services. Medicare fee-for-service provider utilization & payment data physician and other supplier public use file: a methodological overview. Accessed February 1, 2021. https://www.cms.gov/research-statistics-data-and-systems/statistics-trends-and-reports/medicare-provider-charge-data/downloads/medicare-physician-and-other-supplier-puf-methodology.pdf

[21] Centers for Medicare & Medicaid Services. Provider data catalog. Accessed February 1, 2021. https://data.cms.gov/provider-data/?redirect=true

[22] Centers for Medicare & Medicaid Services. 2021 ASP drug pricing files. Accessed February 10, 2021. https://www.cms.gov/medicare/medicare-part-b-drug-average-sales-price/2021-asp-drug-pricing-files

