# Supplementary material for: Association of Multiple Hospital Affiliations With Clinician Service Use, Breadth of Procedures, and Costs
Source: JAMA Netw Open. 2021 Dec 16;4(12):e2139169. doi: 10.1001/jamanetworkopen.2021.39169 (PMC8678686; doi:10.1001/jamanetworkopen.2021.39169)
Supplement: Supplement. — eAppendix 1. Statistical Analysis Additional Details eTable 1. Clinicians Primary Specialty Tabulations for the Medical Service Sample eTable 2. Clinicians Primary Specialty Tabulations for the Drug Service Sample eTable 3. Poisson Model Estimates Across Count Outcome Measure eTable 4. Regression Estimates Across Count Outcome Measure, With Quadratic Term Included to Evaluate Monotonicity of Association eTable 5. Regression Estimates Across Medical Outcome Measure eTable 6. Regression Estimates Across Drug Outcome Measure eTable 7. Summary Statistics for Outcome Measures, Affiliation Measures, and Control Variables Across Medical Services Sample, and Drug Services Sample eTable 8. Summary Statistics for Outcome Measures, Across Medical and Drug Service Samples, by Number of Clinician Hospital Affiliations eAppendix 2. Heterogenous Treatment Effects eTable 9. Heterogenous Treatment Association Estimates Across Internal Medicine (IM), Family/General Practice (FP) and Nurse Practitioners (NP) When Using the Dichotomous Multihospital Affiliation Measure eTable 10. Heterogenous Treatment Association Estimates Across Internal Medicine (IM), Family/General Practice (FP) and Nurse Practitioners (NP) for the Extensive and Intensive Margin Results for Medical Services eTable 11. Heterogenous Treatment Association Estimates Across Internal Medicine (IM), Family/General Practice (FP) and Nurse Practitioners (NP) for the Extensive and Intensive Margin Results for Drug Services eTable 12. Pooled Regression Estimates Across Medical Outcome Measure [file jamanetwopen-e2139169-s001.pdf]

Supplementary Online Content

Linde S, Beilfuss S. Association of multiple hospital affiliations with clinician service use, breadth of procedures, and costs. *JAMA Netw Open.* 2021;4(12):e2139169. doi:10.1001/jamanetworkopen.2021.39169

eAppendix 1. Statistical Analysis Additional Details
eTable 1. Clinicians Primary Specialty Tabulations for the Medical Service Sample
eTable 2. Clinicians Primary Specialty Tabulations for the Drug Service Sample
eTable 3. Poisson Model Estimates Across Count Outcome Measure
eTable 4. Regression Estimates Across Count Outcome Measure, With Quadratic Term Included to Evaluate Monotonicity of Association
eTable 5. Regression Estimates Across Medical Outcome Measure
eTable 6. Regression Estimates Across Drug Outcome Measure
eTable 7. Summary Statistics for Outcome Measures, Affiliation Measures, and Control Variables Across Medical Services Sample, and Drug Services Sample
eTable 8. Summary Statistics for Outcome Measures, Across Medical and Drug Service Samples, by Number of Clinician Hospital Affiliations
eAppendix 2. Heterogenous Treatment Effects
eTable 9. Heterogenous Treatment Association Estimates Across Internal Medicine (IM), Family/General Practice (FP) and Nurse Practitioners (NP) When Using the Dichotomous Multihospital Affiliation Measure
eTable 10. Heterogenous Treatment Association Estimates Across Internal Medicine (IM), Family/General Practice (FP) and Nurse Practitioners (NP) for the Extensive and Intensive Margin Results for Medical Services
eTable 11. Heterogenous Treatment Association Estimates Across Internal Medicine (IM), Family/General Practice (FP) and Nurse Practitioners (NP) for the Extensive and Intensive Margin Results for Drug Services
eTable 12. Pooled Regression Estimates Across Medical Outcome Measure

This supplementary material has been provided by the authors to give readers additional information about their work.

© 2021 Linde S et al. *JAMA Network Open.*

eAppendix 1. Statistical Analysis Additional Details

As noted within the main text, we use panel data regression methods in order to estimate the effect of clinician hospital affiliations on the service breath, procedure breadth, and costs. Because of the inherent limitations of observational study designs, we take the following precautions in order to decrease concerns of potential confounding due to unobserved variables. First, we control for a rich set of clinician, patient pool and volume features. Second, by employing a fixed effects specification we control for clinician and year fixed effects – these allow us to control for any unobserved clinician (or year) differences that might affect both our hospital affiliation measure, as well as our outcome measures. As such, our specification ameliorates concerns pertaining to clinician selection into multi-hospital affiliation based on either observable or unobserved clinician characteristics. In terms of our regression model specification, this is given by:

$$ln(Y_{it}) = \alpha + \tau_1 MultiHospitalAffiliated_{it} + \tau_2 ACOAffiliation_{it} + \boldsymbol{\beta X} + \boldsymbol{\phi_i} + \boldsymbol{\delta_t} + \epsilon_{it}. \text{ (1)}$$

In Equation (1), $ln(Y_{it})$ represents our clinician specific outcome measure of interest in log form, $MultiHospitalAffiliated_{it}$ is a dummy variable indicating whether clinician *i* is affiliated with more than one hospital in year *t,* and $ACOAffiliation_{it}$ is an indicator variable of whether or not clinician *i* is affiliated with a Medicare ACO in year *t.* Furthermore, $\boldsymbol{X}$ is a vector of our other covariates (previously described), ϕ_i captures the clinician specific fixed effects, and $\boldsymbol{\delta_t}$ denotes the time specific fixed effects. Thus, the marginal effect of hospital affiliation upon the outcome is identified using within clinician variation conditional on the additional control variables. Within our secondary

© 2021 Linde S et al. *JAMA Network Open.*

extensive- and intensive-margin analysis we replace the variable "$MultiHospitalAffiliated_{it}$" with $HospitalAffiliations_{it}$ as our main exposure variable. This variable captures the number of hospital affiliations that clinician *i* has in year *t*.

© 2021 Linde S et al. *JAMA Network Open.*

eTable 1. Clinicians Primary Specialty Tabulations for the Medical Service Sample

	Freq.	Percent	Cum.
ADDICTION MEDICINE	220	0.02	0.02
ADVANCED HEART FAILURE AND TRANSPLANT CARDIOLOGY	182	0.01	0.03
ALLERGY/IMMUNOLOGY	3684	0.26	0.29
ANESTHESIOLOGY	67386	4.80	5.09
ANESTHESIOLOGY ASSISTANT	3359	0.24	5.33
AUDIOLOGIST	10614	0.76	6.08
CARDIAC ELECTROPHYSIOLOGY	3286	0.23	6.32
CARDIAC SURGERY	2507	0.18	6.49
CARDIOVASCULAR DISEASE (CARDIOLOGY)	33496	2.38	8.88
CERTIFIED NURSE MIDWIFE	1335	0.10	8.97
CERTIFIED REGISTERED NURSE ANESTHETIST	73053	5.20	14.17
CHIROPRACTIC	49572	3.53	17.70
CLINICAL NURSE SPECIALIST	2769	0.20	17.90
CLINICAL PSYCHOLOGIST	12736	0.91	18.80
CLINICAL SOCIAL WORKER	10591	0.75	19.56
COLORECTAL SURGERY (PROCTOLOGY)	2613	0.19	19.74
CRITICAL CARE (INTENSIVISTS)	5478	0.39	20.13
DENTIST	11	0.00	20.14
DERMATOLOGY	15067	1.07	21.21
DIAGNOSTIC RADIOLOGY	50159	3.57	24.78
EMERGENCY MEDICINE	29270	2.08	26.86
ENDOCRINOLOGY	7789	0.55	27.42
FAMILY PRACTICE	128184	9.12	36.54
GASTROENTEROLOGY	19169	1.36	37.90
GENERAL PRACTICE	6509	0.46	38.37
GENERAL SURGERY	33049	2.35	40.72
GERIATRIC MEDICINE	2749	0.20	40.91
GERIATRIC PSYCHIATRY	331	0.02	40.94
GYNECOLOGICAL ONCOLOGY	1519	0.11	41.05
HAND SURGERY	2542	0.18	41.23
HEMATOLOGY	1223	0.09	41.31
HEMATOLOGY/ONCOLOGY	14156	1.01	42.32
HEMATOPOIETIC CELL TRANSPLANTATION AND CELLULAR TH	9	0.00	42.32
HOSPICE/PALLIATIVE CARE	1750	0.12	42.45
HOSPITALIST	6825	0.49	42.93
INFECTIOUS DISEASE	7784	0.55	43.49
INTERNAL MEDICINE	161234	11.48	54.96
INTERVENTIONAL CARDIOLOGY	4606	0.33	55.29
INTERVENTIONAL PAIN MANAGEMENT	2790	0.20	55.49
INTERVENTIONAL RADIOLOGY	2686	0.19	55.68
MAXILLOFACIAL SURGERY	1055	0.08	55.76
MEDICAL ONCOLOGY	5245	0.37	56.13
MEDICAL TOXICOLOGY	7	0.00	56.13
NEPHROLOGY	12782	0.91	57.04
NEUROLOGY	21630	1.54	58.58
NEUROPSYCHIATRY	195	0.01	58.59
NEUROSURGERY	8129	0.58	59.17
NUCLEAR MEDICINE	908	0.06	59.24

© 2021 Linde S et al. *JAMA Network Open.*

NURSE PRACTITIONER	129739	9.23	68.47
OBSTETRICS/GYNECOLOGY	35825	2.55	71.02
OCCUPATIONAL THERAPY	5893	0.42	71.44
OPHTHALMOLOGY	27533	1.96	73.40
OPTOMETRY	51437	3.66	77.06
ORAL SURGERY	756	0.05	77.11
ORAL SURGERY (DENTIST ONLY)	750	0.05	77.17
ORTHOPEDIC SURGERY	35451	2.52	79.69
OSTEOPATHIC MANIPULATIVE MEDICINE	777	0.06	79.75
OTOLARYNGOLOGY	12337	0.88	80.62
PAIN MANAGEMENT	3356	0.24	80.86
PATHOLOGY	21332	1.52	82.38
PEDIATRIC MEDICINE	1925	0.14	82.52
PERIPHERAL VASCULAR DISEASE	103	0.01	82.53
PHYSICAL MEDICINE AND REHABILITATION	11538	0.82	83.35
PHYSICAL THERAPY	72965	5.19	88.54
PHYSICIAN ASSISTANT	42029	2.99	91.53
PLASTIC AND RECONSTRUCTIVE SURGERY	4895	0.35	91.88
PODIATRY	21766	1.55	93.43
PREVENTATIVE MEDICINE	369	0.03	93.46
PSYCHIATRY	31801	2.26	95.72
PULMONARY DISEASE	14847	1.06	96.78
RADIATION ONCOLOGY	7451	0.53	97.31
REGISTERED DIETITIAN OR NUTRITION PROFESSIONAL	2207	0.16	97.46
RHEUMATOLOGY	7552	0.54	98.00
SLEEP LABORATORY/MEDICINE	291	0.02	98.02
SLEEP MEDICINE	353	0.03	98.05
SPEECH LANGUAGE PATHOLOGIST	827	0.06	98.10
SPORTS MEDICINE	1782	0.13	98.23
SURGICAL ONCOLOGY	1223	0.09	98.32
THORACIC SURGERY	3960	0.28	98.60
UNDEFINED NON-PHYSICIAN TYPE (SPECIFY)	3	0.00	98.60
UNDEFINED PHYSICIAN TYPE (SPECIFY)	367	0.03	98.63
UROLOGY	13770	0.98	99.61
VASCULAR SURGERY	5521	0.39	100.00
Total	1404974	100.00	

© 2021 Linde S et al. *JAMA Network Open.*

eTable 2. Clinicians Primary Specialty Tabulations for the Drug Service Sample

	Freq.	Percent	Cum.
ADDICTION MEDICINE	23	0.01	0.01
ADVANCED HEART FAILURE AND TRANSPLANT CARDIOLOGY	12	0.00	0.01
ALLERGY/IMMUNOLOGY	2375	0.58	0.58
ANESTHESIOLOGY	2642	0.64	1.22
CARDIAC ELECTROPHYSIOLOGY	337	0.08	1.31
CARDIAC SURGERY	43	0.01	1.32
CARDIOVASCULAR DISEASE (CARDIOLOGY)	13453	3.26	4.58
CERTIFIED NURSE MIDWIFE	18	0.00	4.58
CERTIFIED REGISTERED NURSE ANESTHETIST	263	0.06	4.64
CHIROPRACTIC	6	0.00	4.65
CLINICAL NURSE SPECIALIST	165	0.04	4.69
CLINICAL SOCIAL WORKER	2	0.00	4.69
COLORECTAL SURGERY (PROCTOLOGY)	14	0.00	4.69
CRITICAL CARE (INTENSIVISTS)	430	0.10	4.79
DERMATOLOGY	9762	2.37	7.16
DIAGNOSTIC RADIOLOGY	15043	3.65	10.81
EMERGENCY MEDICINE	4683	1.13	11.94
ENDOCRINOLOGY	3034	0.74	12.68
FAMILY PRACTICE	96992	23.50	36.18
GASTROENTEROLOGY	1877	0.45	36.64
GENERAL PRACTICE	3705	0.90	37.53
GENERAL SURGERY	724	0.18	37.71
GERIATRIC MEDICINE	1042	0.25	37.96
GERIATRIC PSYCHIATRY	3	0.00	37.96
GYNECOLOGICAL ONCOLOGY	189	0.05	38.01
HAND SURGERY	2229	0.54	38.55
HEMATOLOGY	282	0.07	38.62
HEMATOLOGY/ONCOLOGY	7013	1.70	40.32
HOSPICE/PALLIATIVE CARE	47	0.01	40.33
HOSPITALIST	118	0.03	40.36
INFECTIOUS DISEASE	2046	0.50	40.85
INTERNAL MEDICINE	74324	18.01	58.86
INTERVENTIONAL CARDIOLOGY	1633	0.40	59.26
INTERVENTIONAL PAIN MANAGEMENT	2156	0.52	59.78
INTERVENTIONAL RADIOLOGY	587	0.14	59.92
MAXILLOFACIAL SURGERY	6	0.00	59.92
MEDICAL ONCOLOGY	2137	0.52	60.44
NEPHROLOGY	4026	0.98	61.42
NEUROLOGY	4839	1.17	62.59
NEUROPSYCHIATRY	18	0.00	62.60
NEUROSURGERY	366	0.09	62.68
NUCLEAR MEDICINE	213	0.05	62.74
NURSE PRACTITIONER	48965	11.87	74.60
OBSTETRICS/GYNECOLOGY	1388	0.34	74.94
OPHTHALMOLOGY	7573	1.84	76.77
OPTOMETRY	6	0.00	76.77
ORAL SURGERY	4	0.00	76.78
ORAL SURGERY (DENTIST ONLY)	4	0.00	76.78

© 2021 Linde S et al. *JAMA Network Open.*

ORTHOPEDIC SURGERY	29296	7.10	83.88
OSTEOPATHIC MANIPULATIVE MEDICINE	309	0.07	83.95
OTOLARYNGOLOGY	1614	0.39	84.34
PAIN MANAGEMENT	2130	0.52	84.86
PATHOLOGY	33	0.01	84.87
PEDIATRIC MEDICINE	780	0.19	85.06
PERIPHERAL VASCULAR DISEASE	18	0.00	85.06
PHYSICAL MEDICINE AND REHABILITATION	5419	1.31	86.37
PHYSICIAN ASSISTANT	17228	4.17	90.55
PLASTIC AND RECONSTRUCTIVE SURGERY	627	0.15	90.70
PODIATRY	11641	2.82	93.52
PREVENTATIVE MEDICINE	175	0.04	93.56
PSYCHIATRY	268	0.06	93.63
PULMONARY DISEASE	6116	1.48	95.11
RADIATION ONCOLOGY	690	0.17	95.28
REGISTERED DIETITIAN OR NUTRITION PROFESSIONAL	3	0.00	95.28
RHEUMATOLOGY	6167	1.49	96.77
SLEEP LABORATORY/MEDICINE	26	0.01	96.78
SLEEP MEDICINE	28	0.01	96.79
SPORTS MEDICINE	1484	0.36	97.15
SURGICAL ONCOLOGY	23	0.01	97.15
THORACIC SURGERY	28	0.01	97.16
UNDEFINED PHYSICIAN TYPE (SPECIFY)	66	0.02	97.17
UROLOGY	11177	2.71	99.88
VASCULAR SURGERY	485	0.12	100.00
Total	412648	100.00	

© 2021 Linde S et al. *JAMA Network Open.*

eTable 3. Poisson Model Estimates Across Count Outcome Measure.
Columns 1-2 capture results for the Medical Services Sample, while columns 3-4 provide results for the Drug Services Sample.

	(1)	(2)	(3)	(4)
	Medical Service Breadth	Medical Procedure Breadth	Drug Service Breadth	Drug Procedure Breadth
Affiliation Measures:				
Multi-Hospital Affiliated	0.050***	0.045***	0.041***	0.011***
	(0.040 - 0.059)	(0.042 - 0.047)	(0.017 - 0.064)	(0.006 - 0.016)
ACO Affiliated	-0.006***	0.008***	-0.002	0.007***
	(-0.010 - -0.002)	(0.007 - 0.010)	(-0.021 - 0.017)	(0.003 - 0.011)
Control Measures:				
Experience	0.002	0.000	-0.000	-0.002
	(-0.002 - 0.006)	(-0.002 - 0.002)	(-0.033 - 0.032)	(-0.008 - 0.003)
EHR User	0.028***	0.015***	0.028***	0.007***
	(0.023 - 0.034)	(0.013 - 0.017)	(0.010 - 0.046)	(0.003 - 0.010)
Avg. Risk Score	-0.034***	-0.004	-0.048	-0.008
	(-0.046 - -0.022)	(-0.009 - 0.001)	(-0.156 - 0.060)	(-0.024 - 0.008)
Avg. Patient Age	0.017***	0.005***	0.017	-0.002***
	(0.015 - 0.019)	(0.004 - 0.006)	(-0.013 - 0.047)	(-0.004 - -0.001)
Number of Female Patients	-0.000	-0.000***	0.000	0.000**
	(-0.001 - 0.000)	(-0.000 - -0.000)	(-0.000 - 0.000)	(0.000 - 0.000)
Number of Medical Patients	0.000	0.000***		
	(-0.000 - 0.001)	(0.000 - 0.000)		
Number of Drug Patients			0.004***	0.001***
			(0.003 - 0.004)	(0.001 - 0.002)
Clinician FEs	Yes	Yes	Yes	Yes
Year FEs	Yes	Yes	Yes	Yes
Observations	879,400	879,400	296,818	296,818
Number of Clinicians	439,700	439,700	148,409	148,409

Robust 95% confidence intervals are reported within the parentheses. Significance is denoted as: *** p<0.01, ** p<0.05, * p<0.1.

Abbreviations: ACO = Accountable Care Organization; Avg. = Average; EHR = Electronic Health Records; FE = Fixed Effects.

© 2021 Linde S et al. *JAMA Network Open.*

eTable 4. Regression Estimates Across Count Outcome Measure, With Quadratic Term Included to Evaluate Monotonicity of Association.
Columns 1-3 capture results for the Medical Services Sample, while columns 4-6 provide results for the Drug Services Sample.

	Medical Services Sample			Drug Services Sample		
	(1) Log	(2) Log	(3) Log	(4) Log	(5) Log	(6) Log
	Medical Services	Medical Procedure Breadth	Total Medical Costs	Drug Services	Drug Procedure Breadth	Total Drug Costs
Affiliation Measures:						
Hospital Affiliation	0.089***	0.063***	0.093***	0.032***	0.010***	0.028***
	(0.084 - 0.094)	(0.059 - 0.066)	(0.088 - 0.099)	(0.018 - 0.046)	(0.003 - 0.016)	(0.013 - 0.043)
Hospital Affiliation^2	-0.007***	-0.006***	-0.008***	-0.003**	-0.001	-0.002*
	(-0.009 - -0.006)	(-0.007 - -0.006)	(-0.009 - -0.006)	(-0.005 - -0.000)	(-0.002 - 0.000)	(-0.005 - 0.000)
Clinician FEs	YES	YES	YES	YES	YES	YES
Year FEs	YES	YES	YES	YES	YES	YES
Observations	1,073,252	1,073,252	1,073,252	358,669	358,669	358,669
R-squared	0.195	0.055	0.168	0.202	0.041	0.221
Number of Clinicians	633,552	633,552	633,552	210,260	210,260	210,260

Robust 95% confidence intervals are reported within the parentheses. Significance is denoted as: *** p<0.01, ** p<0.05, * p<0.1.

Abbreviations: ACO = Accountable Care Organization; Avg. = Average; EHR = Electronic Health Records; FE = Fixed Effects.

© 2021 Linde S et al. *JAMA Network Open.*

eTable 5. Regression Estimates Across Medical Outcome Measure.
Columns 1-3 capture results for the full sample (Number of Hospital Affiliations ≥ 0), while columns 4-6 provide results for the sample when we condition on clinicians having at least one hospital affiliation.

	Number of Hospital Affiliations ≥ 0			Number of Hospital Affiliations ≥ 1		
	(1) Log	(2) Log	(3) Log	(4) Log	(5) Log	(6) Log
	Medical Services	Medical Procedure Breadth	Total Medical Costs	Medical Services	Medical Procedure Breadth	Total Medical Costs
Affiliation Measures:						
Hospital Affiliations	0.056***	0.033***	0.058***	0.049***	0.028***	0.052***
	(0.051 - 0.062)	(0.031 - 0.035)	(0.053 - 0.064)	(0.042 - 0.056)	(0.026 - 0.030)	(0.045 - 0.058)
ACO Affiliated	0.001	0.012***	0.000	0.001	0.011***	-0.000
	(-0.002 - 0.004)	(0.010 - 0.014)	(-0.003 - 0.004)	(-0.002 - 0.004)	(0.008 - 0.013)	(-0.003 - 0.003)
Control Measures:						
Experience	0.000	-0.000	-0.000	0.001	-0.001	0.001
	(-0.002 - 0.003)	(-0.002 - 0.001)	(-0.003 - 0.002)	(-0.001 - 0.004)	(-0.003 - 0.001)	(-0.002 - 0.004)
EHR User	0.047***	0.017***	0.043***	0.045***	0.017***	0.039***
	(0.044 - 0.051)	(0.015 - 0.019)	(0.039 - 0.046)	(0.041 - 0.049)	(0.015 - 0.019)	(0.035 - 0.043)
Avg. Risk Score	-0.004	-0.002	0.025***	-0.012***	-0.005*	0.016***
	(-0.012 - 0.003)	(-0.007 - 0.002)	(0.017 - 0.033)	(-0.019 - -0.004)	(-0.010 - 0.000)	(0.008 - 0.024)
Avg. Patient Age	0.017***	0.004***	0.018***	0.018***	0.004***	0.019***
	(0.015 - 0.018)	(0.003 - 0.005)	(0.016 - 0.019)	(0.016 - 0.019)	(0.003 - 0.005)	(0.017 - 0.020)
Number of Female Patients	-0.001*	-0.000	-0.000	-0.001***	-0.000***	-0.001**
	(-0.001 - 0.000)	(-0.000 - 0.000)	(-0.001 - 0.000)	(-0.001 - -0.000)	(-0.000 - -0.000)	(-0.001 - -0.000)
Number of Medical Patients	0.001***	0.000***	0.001***	0.001***	0.000***	0.001***

© 2021 Linde S et al. *JAMA Network Open.*

	(0.001 - 0.002)	(0.000 - 0.000)	(0.001 - 0.001)	(0.001 - 0.002)	(0.000 - 0.000)	(0.001 - 0.001)
Clinician FEs	Yes	Yes	Yes	Yes	Yes	Yes
Year FEs	Yes	Yes	Yes	Yes	Yes	Yes
Observations	1,404,974	1,404,974	1,404,974	1,073,252	1,073,252	1,073,252
R-squared	0.169	0.044	0.149	0.195	0.054	0.167
Number of Clinicians	824,512	824,512	824,512	633,552	633,552	633,552

Robust 95% confidence intervals are reported within the parentheses. Significance is denoted as: *** p<0.01, ** p<0.05, * p<0.1.

Abbreviations: ACO = Accountable Care Organization; Avg. = Average; EHR = Electronic Health Records; FE = Fixed Effects.

© 2021 Linde S et al. *JAMA Network Open.*

eTable 6. Regression Estimates Across Drug Outcome Measure.
Columns 1-3 capture results for the full sample (Number of Hospital Affiliations ≥ 0), while columns 4-6 provide results for the sample when we condition on clinicians having at least one hospital affiliation.

	Number of Hospital Affiliations ≥ 0			Number of Hospital Affiliations ≥ 1		
	(1)	(2)	(3)	(4)	(5)	(6)
	Drug Services	Drug Procedure Breadth	Total Drug Costs	Drug Services	Drug Procedure Breadth	Total Drug Costs
Affiliation Measures:						
Hospital Affiliations	0.022***	0.007***	0.022***	0.017***	0.004***	0.015***
	(0.018 - 0.027)	(0.005 - 0.009)	(0.017 - 0.027)	(0.013 - 0.022)	(0.002 - 0.007)	(0.010 - 0.019)
ACO Affiliated	0.006	0.009***	0.016***	0.003	0.008***	0.011**
	(-0.003 - 0.014)	(0.005 - 0.013)	(0.007 - 0.025)	(-0.005 - 0.012)	(0.004 - 0.012)	(0.002 - 0.020)
Control Measures:						
Experience	0.003	-0.001	-0.012**	0.005	-0.003	-0.010
	(-0.005 - 0.012)	(-0.006 - 0.003)	(-0.023 - -0.000)	(-0.004 - 0.014)	(-0.008 - 0.003)	(-0.022 - 0.002)
EHR User	0.042***	0.008***	0.034***	0.039***	0.006***	0.028***
	(0.035 - 0.050)	(0.004 - 0.012)	(0.026 - 0.042)	(0.031 - 0.046)	(0.002 - 0.010)	(0.020 - 0.037)
Avg. Risk Score	-0.101***	-0.015**	-0.135***	-0.118***	-0.022***	-0.130***
	(-0.130 - -0.072)	(-0.028 - -0.002)	(-0.167 - -0.104)	(-0.150 - -0.087)	(-0.036 - -0.008)	(-0.164 - -0.096)
Avg. Patient Age	0.003*	-0.004***	0.014***	0.002	-0.003***	0.010***
	(-0.000 - 0.007)	(-0.005 - -0.002)	(0.010 - 0.018)	(-0.002 - 0.007)	(-0.005 - -0.001)	(0.006 - 0.014)
Number of Female Patients	0.000**	0.000***	0.000	0.000**	0.000***	0.000**
	(0.000 - 0.000)	(0.000 - 0.000)	(-0.000 - 0.000)	(0.000 - 0.000)	(0.000 - 0.000)	(0.000 - 0.000)
Number of Drug Patients	0.008***	0.002***	0.009***	0.008***	0.002***	0.009***

	(0.007 - 0.008)	(0.001 - 0.002)	(0.008 - 0.009)	(0.007 - 0.008)	(0.001 - 0.002)	(0.008 - 0.009)
Clinician FEs	Yes	Yes	Yes	Yes	Yes	Yes
Year FEs	Yes	Yes	Yes	Yes	Yes	Yes
Observations	412,648	412,648	412,648	358,669	358,669	358,669
R-squared	0.200	0.041	0.208	0.202	0.041	0.221
Number of Clinicians	241,335	241,335	241,335	210,260	210,260	210,260

Robust 95% confidence intervals are reported within the parentheses. Significance is denoted as: *** p<0.01, ** p<0.05, * p<0.1.

Abbreviations: ACO = Accountable Care Organization; Avg. = Average; EHR = Electronic Health Records; FE = Fixed Effects.

© 2021 Linde S et al. *JAMA Network Open.*

eTable 7. Summary Statistics for Outcome Measures, Affiliation Measures, and Control Variables Across: (i) Medical Services Sample, and (ii) Drug Services Sample. These Samples Include both Affiliated and Non-Affiliated Clinicians.

	**Medical Services Sample**		**Drug Services Sample**	
Variable	Mean	(SD)	Mean	(SD)
Medical Outcome Measures:				
Medical Services	1,494.29	(2,941.25)	NA	NA
Medical Procedures Breadth	34.42	(34.28)	NA	NA
Total Medical Costs	81,637.13	(132,271.39)	NA	NA
Drug Outcome Measures:				
Drug Services	NA	NA	5,008.11	(26,482.7)
Drug Procedures Breadth	NA	NA	7.69	(9.37)
Total Drug Costs	NA	NA	57,937.53	(299,806.17)
Affiliation Measures:				
Hospital Affiliations	1.69	(1.52)	2.22	(1.56)
Multi-Hospital Affiliated (%)	58%	(49%)	7%	(46%)
ACO Affiliated (%)	23%	(42%)	28%	(45%)
Control Variables:				
Experience	20.27	(12.54)	22.03	(12.34)
EHR User (%)	26%	(44%)	46%	(5%)
Avg. Risk Score	1.64	(0.78)	1.39	(.57)
Avg. Patient Age	71.03	(5.44)	72.14	(3.72)
Number of Female Patients	220.3	(367.74)	297.54	(382.84)
Number of Medical Patients	384.12	(621.73)	NA	NA
Number of Drug Patients	NA	NA	93.77	(108.5)
Number of Observations	*1,404,974*		*412,648*	

Abbreviations: ACO = Accountable Care Organization; Avg. = Average; EHR = Electronic Health Records.

© 2021 Linde S et al. *JAMA Network Open.*

eTable 8. Summary Statistics for Outcome Measures, Across Medical and Drug Service Samples, by Number of Clinician Hospital Affiliations

	Medical Services Sample				Drug Services Sample			
Hospital Affiliations	Sample (%)	Medical Services	Medical Procedure Breadth	Total Medical Costs	Sample (%)	Drug Services	Drug Procedure Breadth	Total Medical Costs
0	23.61	1146.74	12.1	43,846.16	13.08	1877.66	5.87	29,189.42
1	32.29	810.53	29.48	51,835.44	25.88	2383	7	44,802.32
2	18.79	1316.18	37.97	79,999.9	22.96	3707.68	7.7	45,338.79
3	10.50	1842.47	44.54	109,318.16	15.61	5679.13	8.34	57,293.23
4	5.90	2299.23	50.06	135,352.28	9.42	7891.4	8.83	74,132.78
5	8.90	3241.36	66.19	176,644.82	13.05	12758.58	9.29	124,071.53
Number of Observations		*1,404,974*				*412,648*		

© 2021 Linde S et al. *JAMA Network Open.*

eAppendix 2. Heterogenous Treatment Effects

eTables 9-11 provide the treatment effect results when we further allow for heterogenous treatment responses across the specialties of: (i) internal medicine, (ii) family/general practice, and (iii) nurse practitioners. These results indicate that there are indeed heterogenous treatment responses to multi-hospital affiliation across these clinician groups, and it furthermore indicates that these heterogeneities appear to be more pronounced for medical than for drug services.

© 2021 Linde S et al. *JAMA Network Open.*

eTable 9. Heterogenous Treatment Association Estimates Across Internal Medicine (IM), Family/General Practice (FP) and Nurse Practitioners (NP) When Using the Dichotomous Multihospital Affiliation Measure

	Medical Services Sample			Drug Services Sample		
	(1) Log	(2) Log	(3) Log	(4) Log	(5) Log	(6) Log
	Medical Services	Medical Procedure Breadth	Total Medical Costs	Drug Services	Drug Procedure Breadth	Total Drug Costs
Affiliation Measures:						
Multi-Hospital Affiliated	0.075***	0.057***	0.078***	0.030***	0.008**	0.018*
	(0.067 - 0.082)	(0.053 - 0.060)	(0.070 - 0.085)	(0.015 - 0.044)	(0.000 - 0.016)	(-0.002 - 0.038)
Multi-Hospital Affiliated*IM	0.024***	-0.006*	0.026***	-0.004	-0.002	0.000
	(0.015 - 0.033)	(-0.012 - 0.000)	(0.017 - 0.035)	(-0.030 - 0.022)	(-0.015 - 0.011)	(-0.027 - 0.027)
Multi-Hospital Affiliated*FP	-0.009	-0.022***	-0.006	0.003	0.001	0.017
	(-0.020 - 0.002)	(-0.029 - -0.015)	(-0.018 - 0.006)	(-0.021 - 0.027)	(-0.010 - 0.012)	(-0.009 - 0.043)
Multi-Hospital Affiliated*NP	0.043***	0.004	0.041***	-0.005	0.012	0.032
	(0.028 - 0.058)	(-0.006 - 0.014)	(0.026 - 0.057)	(-0.043 - 0.034)	(-0.005 - 0.029)	(-0.010 - 0.074)
Controls	Yes	Yes	Yes	Yes	Yes	Yes
Clinician FEs	Yes	Yes	Yes	Yes	Yes	Yes
Year FEs	Yes	Yes	Yes	Yes	Yes	Yes
Observations	1,073,252	1,073,252	1,073,252	358,669	358,669	358,669
R-squared	0.191	0.052	0.163	0.202	0.041	0.221
Number of Clinicians	633,552	633,552	633,552	210,260	210,260	210,260

Robust 95% confidence intervals are reported within the parentheses. Significance is denoted as: *** p<0.01, ** p<0.05, * p<0.1.

Abbreviations: FE = Fixed Effects; FP = Family/General Practice; IM = Internal Medicine; NP = Nurse Practitioner.

© 2021 Linde S et al. *JAMA Network Open.*

eTable 10. Heterogenous Treatment Association Estimates Across Internal Medicine (IM), Family/General Practice (FP) and Nurse Practitioners (NP) for the Extensive and Intensive Margin Results for Medical Services

	Number of Hospital Affiliations = 0 or 1			Number of Hospital Affiliations ≥ 1		
	Log Medical Services	Log Medical Procedure Breadth	Log Total Medical Costs	Log Medical Services	Log Medical Procedure Breadth	Log Total Medical Costs
Affiliation Measures:						
Hospital Affiliations	0.076***	0.091***	0.084***	0.045***	0.029***	0.047***
	(0.065 - 0.087)	(0.084 - 0.098)	(0.073 - 0.095)	(0.037 - 0.053)	(0.026 - 0.032)	(0.039 - 0.054)
Hospital Affiliations*IM	0.029*	-0.048***	0.024	0.009***	-0.004***	0.013***
	(-0.001 - 0.059)	(-0.067 - -0.029)	(-0.007 - 0.054)	(0.005 - 0.014)	(-0.006 - -0.001)	(0.009 - 0.017)
Hospital Affiliations*FP	-0.002	-0.063***	-0.005	-0.007*	-0.009***	-0.005
	(-0.024 - 0.020)	(-0.079 - -0.047)	(-0.029 - 0.018)	(-0.014 - 0.001)	(-0.012 - -0.005)	(-0.012 - 0.002)
Hospital Affiliations*NP	0.046***	-0.014	0.035***	0.042***	0.008**	0.042***
	(0.021 - 0.070)	(-0.031 - 0.003)	(0.009 - 0.061)	(0.032 - 0.051)	(0.002 - 0.014)	(0.032 - 0.051)
Controls	Yes	Yes	Yes	Yes	Yes	Yes
Clinician FEs	Yes	Yes	Yes	Yes	Yes	Yes
Year FEs	Yes	Yes	Yes	Yes	Yes	Yes
Observations	785,437	785,437	785,437	1,073,252	1,073,252	1,073,252
R-squared	0.141	0.029	0.131	0.195	0.054	0.168
Number of Clinicians	509,564	509,564	509,564	633,552	633,552	633,552

Robust 95% confidence intervals are reported within the parentheses. Significance is denoted as: *** p<0.01, ** p<0.05, * p<0.1.

Abbreviations: FE = Fixed Effects; FP = Family/General Practice; IM = Internal Medicine; NP = Nurse Practitioner.

© 2021 Linde S et al. *JAMA Network Open.*

eTable 11. Heterogenous Treatment Association Estimates Across Internal Medicine (IM), Family/General Practice (FP) and Nurse Practitioners (NP) for the Extensive and Intensive Margin Results for Drug Services

	Number of Hospital Affiliations = 0 or 1			Number of Hospital Affiliations ≥ 1		
	Log Drug Services	Log Drug Procedure Breadth	Log Total Drug Costs	Log Drug Services	Log Drug Procedure Breadth	Log Total Drug Costs
Affiliation Measures:						
Hospital Affiliations	0.043***	0.013*	0.020	0.015***	0.004**	0.009**
	(0.014 - 0.072)	(-0.001 - 0.027)	(-0.022 - 0.062)	(0.009 - 0.022)	(0.000 - 0.007)	(0.001 - 0.016)
Hospital Affiliations*IM	0.014	0.016	0.076**	-0.001	-0.001	0.002
	(-0.046 - 0.074)	(-0.013 - 0.044)	(0.013 - 0.139)	(-0.011 - 0.010)	(-0.006 - 0.004)	(-0.009 - 0.013)
Hospital Affiliations*FP	0.016	0.010	0.058**	0.004	0.002	0.011**
	(-0.032 - 0.064)	(-0.011 - 0.032)	(0.003 - 0.114)	(-0.006 - 0.015)	(-0.003 - 0.007)	(0.000 - 0.022)
Hospital Affiliations*NP	0.026	0.022*	0.040	0.013	0.006	0.040***
	(-0.028 - 0.079)	(-0.004 - 0.048)	(-0.028 - 0.109)	(-0.009 - 0.036)	(-0.004 - 0.016)	(0.016 - 0.064)
Controls	Yes	Yes	Yes	Yes	Yes	Yes
Clinician FEs	Yes	Yes	Yes	Yes	Yes	Yes
Year FEs	Yes	Yes	Yes	Yes	Yes	Yes
Observations	160,783	160,783	160,783	358,669	358,669	358,669
R-squared	0.183	0.040	0.162	0.202	0.042	0.221
Number of Clinicians	110,529	110,529	110,529	210,260	210,260	210,260

Robust 95% confidence intervals are reported within the parentheses. Significance is denoted as: *** p<0.01, ** p<0.05, * p<0.1.

Abbreviations: FE = Fixed Effects; FP = Family/General Practice; IM = Internal Medicine; NP = Nurse Practitioner.

© 2021 Linde S et al. *JAMA Network Open.*

eTable 12. Pooled Regression Estimates Across Medical Outcome Measure. Columns 1-3 capture results for the Medical Services Sample, while columns 4-6 provide results for the Drug Services Sample.

	Medical Services Sample			Drug Services Sample		
	(1) Log	(2) Log	(3) Log	(4) Log	(5) Log	(6) Log
	Medical Services	Medical Procedure Breadth	Total Medical Costs	Drug Services	Drug Procedure Breadth	Total Drug Costs
Affiliation Measures:						
Multi-Hospital Affiliated	0.368***	0.182***	0.368***	0.193***	0.021***	0.246***
	(0.354 - 0.381)	(0.177 - 0.188)	(0.356 - 0.381)	(0.183 - 0.204)	(0.016 - 0.025)	(0.234 - 0.257)
ACO Affiliated	-0.125***	-0.013***	-0.129***	-0.109***	0.050***	0.190***
	(-0.129 - -0.121)	(-0.015 - -0.010)	(-0.133 - -0.126)	(-0.119 - -0.100)	(0.047 - 0.054)	(0.180 - 0.200)
Observations	1,073,252	1,073,252	1,073,252	358,669	358,669	358,669
R-squared	0.489	0.273	0.428	0.322	0.179	0.303
Year FE	YES	YES	YES	YES	YES	YES
Specialty FE	YES	YES	YES	YES	YES	YES

Robust 95% confidence intervals are reported within the parentheses. Significance is denoted as: *** p<0.01, ** p<0.05, * p<0.1.

Abbreviations: ACO = Accountable Care Organization; FE = Fixed Effects.

© 2021 Linde S et al. *JAMA Network Open.*
